# Adverse outcomes associated with rapid linear and non-linear patterns of chronic kidney disease progression

**DOI:** 10.1186/s12882-021-02282-5

**Published:** 2021-03-06

**Authors:** Ibrahim Ali, Rajkumar Chinnadurai, Sara T. Ibrahim, Philip A. Kalra

**Affiliations:** 1 Department of Renal Medicine, Salford Royal NHS Foundation Trust, Stott Lane, Salford, M6 8HD UK; 2 Division of Cardiovascular Sciences, University of Manchester, Manchester, M13 9PL UK; 3 Department of Internal Medicine and Nephrology, Faculty of Medicine, Alexandria University, Alexandria, Egypt

**Keywords:** Chronic kidney disease, CKD, Linear, Non-linear, Progression, End-stage renal disease, ESRD

## Abstract

**Background:**

Patients with rapidly declining renal function face the dual threat of end-stage renal disease (ESRD) and mortality prior to ESRD. What is less well characterised is whether the pattern of the renal trajectory, linear or non-linear, unmasks subgroups of rapidly progressing patients that face adverse outcomes in a differential manner.

**Methods:**

An individual eGFR slope was applied to all outpatient estimated glomerular filtration rate (eGFR) values for each patient in the Salford Kidney Study from 2002 to 2018 who had at least 2 years follow-up, ≥4 eGFR values and baseline eGFR 15 to < 60 ml/min/1.73m^2^. Rapid progression was defined as an annual eGFR slope of ≤ − 3 ml/min/1.73m^2^/yr and patients were categorised as linear or non-linear progressors based on the nature of their eGFR-time graphs. A Fine-Gray competing risk hazard model was used to determine factors associated with progression to ESRD and with mortality prior to ESRD. Cumulative incidence function curves highlighted differences in outcomes between linear and non-linear patients.

**Results:**

There were 211 rapidly deteriorating patients with linear eGFR trajectories and 61 rapid non-linear patients in the study cohort. Factors associated with ESRD included younger age, male gender, lower baseline eGFR and higher serum phosphate, whilst older age, history of myocardial infarction and anaemia predicted mortality prior to ESRD. Over a median follow-up of 3.7 years, linear progressors reached ESRD sooner whilst those with non-linear progression faced significantly higher rates of mortality prior to ESRD.

**Conclusions:**

Patients with rapid eGFR decline have high rates of adverse outcomes that are differentially expressed in those progressing linearly and non-linearly as a result of differing phenotypic profiles. Consequently, addressing individual risk factor profiles is important to deliver optimal personalised patient care.

**Supplementary Information:**

The online version contains supplementary material available at 10.1186/s12882-021-02282-5.

## Background

Patients with chronic kidney disease (CKD) that experience rapidly declining renal function are at increased risk of adverse outcomes, including increased risk of end-stage renal disease (ESRD) as well as increased risk of mortality prior to ESRD [1]. Over the past decade, there has been growing attention to not only considering the slope of renal function change, as defined by annual changes in estimated glomerular filtration rate (eGFR) [2], but also the pattern of the eGFR trajectory on patient outcomes [3, 4], especially given that patients with CKD progress in ways other than in a simply linear manner [5].

Extending our understanding of how the eGFR slope and trajectory impacts patient outcomes could help to refine current risk prediction tools to stratify high-risk patients more accurately. This would help improve the communication of risk to patients and help shape management strategies, including earlier targeted treatment in an attempt to assuage future harm [6].

Whilst several studies have recognised the determinants of rapid progression, what is less well known is whether the pattern of rapid CKD progression, be it linear or non-linear, has an impact on patient outcomes. We therefore undertook this study to 1) identify the predictive factors of rapid progression in a cohort of patients progressing in a linear and non-linear pattern; 2) to examine how the pattern of rapid progression affects outcomes of ESRD and mortality prior to ESRD, and in doing so, 3) identify whether there are subgroups and phenotypic differences between linear and non-linear progressors that could enlighten specific approaches to patient management.

## Methods

### Patient population

Patients were drawn from the Salford Kidney Study (SKS), an ongoing observational cohort study, which since 2002 has been recruiting patients aged ≥18 years old with non-dialysis CKD who have been referred to the renal services at Salford Royal NHS Foundation Trust in the United Kingdom. The SKS received ethical approval from the North West Greater Manchester South Research Ethics Committee (REC15/NW/0818). Written informed consent was obtained from all patients. The methods described herein were carried out in accordance with relevant guidelines and regulations of the SKS.

### Baseline characteristics

Patient characteristics were measured at the point of recruitment into the SKS. They include patient demographics (age, gender, ethnicity, history of past or current smoking); past medical history (primary renal disease, history of hypertension (HTN), diabetes mellitus (DM), myocardial infarction (MI), peripheral vascular disease (PVD), stroke and heart failure (HF)); medication history (use of angiotensin converting enzyme inhibitor (ACEi), angiotensin receptor blockers (ARB) and statins); and laboratory measurements (serum creatinine, eGFR calculated using the CKD epidemiology collaboration (CKD-EPI) equation, bicarbonate, urea, calcium, phosphate, albumin, haemoglobin and urine protein:creatinine ratio (uPCR), which was categorised into albuminuria grades of A1, A2 and A3 based on values of < 15 g/mol, 15-50 g/mol and > 50 g/mol respectively) [7].

### Assembling the study cohort

**Fig. 1:**
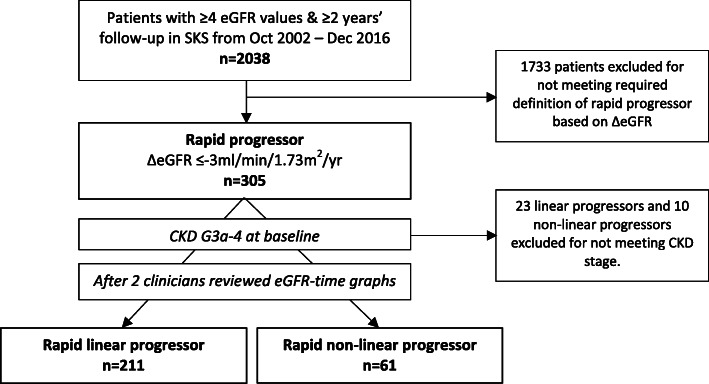
Assembling the study cohort. Abbreviations: SKS (Salford Kidney Study)

Patient selection into this study is shown in Fig. 1. All the outpatient eGFR values, performed as part of routine renal care and accessed via the hospital’s electronic patient record, were used to calculate the delta (Δ) eGFR slope for each patient using linear regression. As a minimum, we required at least 4 eGFR values over 2 years follow-up for the ΔeGFR to be ascertained for each patient. Patients with a ΔeGFR≤ − 3 ml/min/1.73m^2^/yr (ie. losing more than 3ml/min/1.73m^2^/yr), a threshold associated with worse outcomes [2], were defined as a rapid progressor, and they had to have baseline CKD G3a-4 (eGFR 15 to < 60 ml/min/1.73m^2^) for study inclusion. To differentiate linear versus non-linear progression, the eGFR-time graphs were visually inspected independently by two clinicians, an approach that has been successfully utilised in previous studies [1, 8], and also quantitively assessed with the coefficient of determination (R^2^).

### Study outcomes

Patient outcomes included ESRD (haemodialysis, peritoneal dialysis, conservative care or pre-emptive transplantation) or mortality prior to ESRD. Outcome events were reviewed until 1st January 2020.

### Statistical analysis

Continuous data is presented as median (± interquartile range) and categorical data as number (percentage). To compare variables between rapid linear and rapid non-linear progressors, Mann-Whitney U test was used for continuous data and chi-squared test for categorical covariates. The Fine-Gray competing risk hazard model [9] was employed to determine the sub-distribution hazard ratios of the factors associated with ESRD or mortality prior to ESRD within the study cohort. The following 20 variables were included in the model: age, gender, SBP, DBP, HTN, DM, smoking, MI, PVD, stroke, HF, ACEi/ARB use, statin use, eGFR, bicarbonate, calcium, phosphate, albumin, Hb and A3 proteinuria. The proportional hazards assumption for each model was assessed by the non-significance of each time-by-variable interaction.

Cumulative incidence function curves were produced comparing the outcomes of ESRD and mortality between linear and non-linear progressors, for which a modified Chi-squared test was used for significance testing [10]. A comparison between linear and non-linear progressors on a composite outcome of either ESRD or mortality was also assessed and visualised as a 1-Kaplan-Meier curve, which used log-rank significance testing. Statistical significance was defined as a *p* value of < 0.05. Analyses were performed using SPSS (Version 25.0) (IBM SPSS, Chicago, IL) licensed to the University of Manchester and R version 4.0.2 (The R Foundation for Statistical Computing Platform).

## Results

### Baseline characteristics

**Fig. 2:**
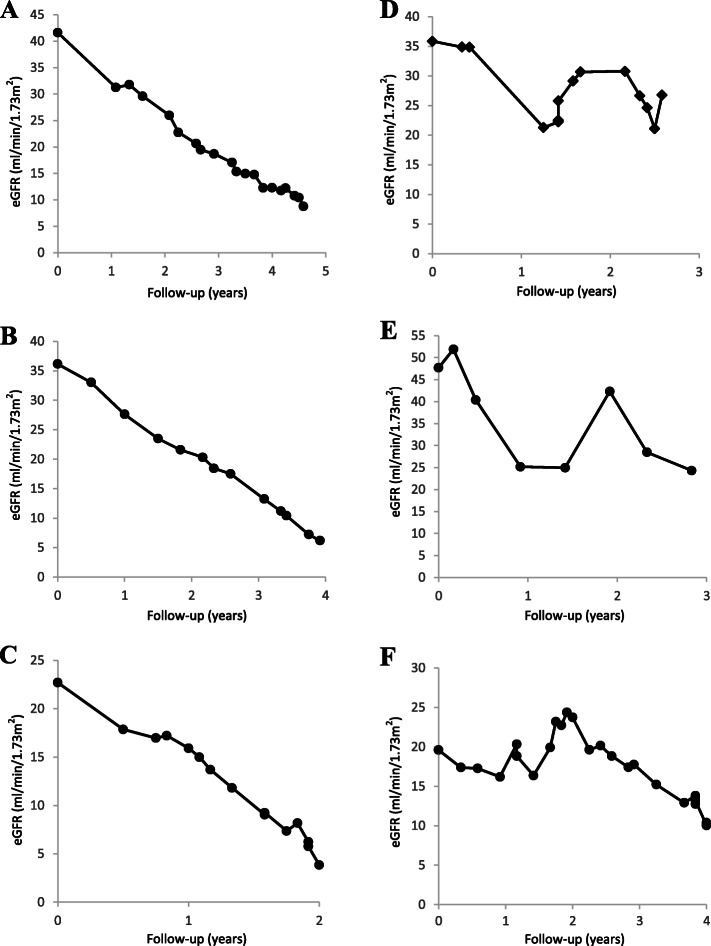
Examples of eGFR-time graphs of linear and non-linear progressors in the study cohort. Graphs A to C highlight examples of rapid linear progression, whilst graphs D to F show rapid non-linear eGFR trajectory

A total of 272 patients met the inclusion criteria of which 211 patients had linear progression and 61 patients had non-linear progression (Table 1). Categorisation as a linear or non-linear progressor was achieved with unanimous agreement between the two clinicians independently reviewing patients’ eGFR-time graphs, illustrative examples of which are shown in Fig. 2. Quantitatively, linear patients had a significantly higher median R^2^ value of 0.91 (0.81–0.95) compared with 0.58 (0.36–0.60) in non-linear progressors, *p*-value < 0.01.

**Table 1:** Baseline characteristics of the study cohort

Variable	All patients (***n*** = 272)	Rapid linear progressor (***n*** = 211)	Rapid non-linear progressor (***n*** = 61)	p-value
Age (years)	58.0 (46.0–70.0)	54.8 (44.9–67.2)	67.4 (58.9–74.5)	**< 0.01**
Men, *n* (%)	134 (49)	104 (49)	30 (49)	0.99
Caucasian, *n* (%)	231 (85)	171 (81)	60 (98)	**< 0.01**
Systolic blood pressure (mmHg)	140 (128–155)	140 (128–154)	140 (129–156)	0.75
Diastolic blood pressure (mmHg)	78 (70–84)	79 (70–84)	76 (70–81)	0.17
Hypertension, *n* (%)	254 (93)	197 (93)	57 (93)	0.98
Diabetes, *n* (%)	90 (33)	59 (28)	31 (51)	**< 0.01**
Past/current smoking history, *n* (%)	174 (64)	132 (63)	42 (69)	0.37
Myocardial infarction, *n* (%)	26 (10)	12 (6)	14 (23)	**< 0.01**
Peripheral vascular disease, *n* (%)	33 (12)	20 (10)	13 (21)	**0.01**
Stroke, *n* (%)	20 (7)	12 (6)	8 (13)	0.05
Heart failure, *n* (%)	27 (10)	13 (6)	14 (23)	**< 0.01**
ACEi/ARB, *n* (%)	197 (72)	158 (75)	39 (64)	0.09
Statin, *n* (%)	158 (58)	120 (57)	38 (62)	0.45
Years follow-up	3.7 (2.8–4.9)	3.9 (2.9–5.0)	3.2 (2.6–4.0)	**< 0.01**
**Primary renal disease**				
Diabetic nephropathy, *n* (%)	67 (25)	46 (22)	21 (34)	**0.04**
ADPKD, *n* (%)	55 (20)	55 (26)	0 (0)	**< 0.01**
Hypertensive nephropathy, *n* (%)	26 (10)	15 (7)	11 (18)	**0.01**
Glomerulonephritis, *n* (%)	34 (13)	32 (15)	2 (3)	**0.01**
Other causes, *n* (%)	66 (24)	45 (21)	21 (23)	**0.04**
Unknown, *n* (%)	24 (9)	18 (9)	6 (10)	0.75
**Laboratory results**				
eGFR-EPI (ml/min/1.73m^2^)	34 (26–41)	34 (26–41)	32 (23–41)	0.36
eGFR measurements per patient, *n*	24 (15–37)	24 (16–36)	21 (11–37)	0.19
ΔGFR (±ml/min/1.73m^2^/yr)	−5.23 (−6.72 to −3.98)	−5.28 (−6.75 to −4.11)	−4.57 (−6.46 to −3.46)	0.06
Bicarbonate (mmol/L)	22.6 (20.4–25.0)	22.5 (20.4–25.0)	23.0 (20.6–25.2)	0.43
Urea (mmol/L)	12.2 (9.8–15.7)	12.0 (9.9–15.4)	13.9 (10.1–17.2)	0.24
Calcium (mmol/L)	2.31 (2.23–2.39)	2.30 (2.22–2.34)	2.32 (2.27–2.40)	0.15
Phosphate (mmol/L)	1.13 (1.02–1.27)	1.15 (1.02–1.28)	1.11 (1.03–1.25)	0.74
Albumin (g/L)	42 (39–44)	41 (39–44)	42 (39–44)	0.99
Total cholesterol/HDL ratio	3.4 (2.7–4.3)	3.5 (2.7–4.5)	3.2 (2.6–4.1)	0.16
Haemoglobin (g/L)	122 (113–133)	123 (114–133)	121 (110–132)	0.57
Urine protein:creatinine ratio (g/mol)	77 (24–244)	87 (26–272)	52 (24–171)	0.16
A1 proteinuria (< 15 g/mol), *n* (%)	33 (12)	26 (12)	7 (12)	0.86
A2 proteinuria (15-50 g/mol), *n* (%)	82 (30)	59 (28)	23 (38)	0.14
A3 proteinuria (> 50 g/mol), *n* (%)	157 (58)	126 (60)	31 (51)	0.22

Continuous data are presented as median (interquartile range) and categorical variables presented as number (percentage)

*P*-values, comparing linear and non-linear groups, were calculated by Mann-Whitney test and Chi-squared test for continuous and categorical data respectively

*Abbreviations*: *ADPKD* (autosomal dominant polycystic kidney disease), *ACEi* (angiotensin-converting enzyme inhibitor), *ARB* (angiotensin receptor blocker), *eGFR-EPI* (eGFR calculated using the EPI-equation)

There were no significant differences in laboratory measures between the two patient groups. Of note, both groups demonstrated poor baseline renal function with a median eGFR of 34 ml/min/1.73m^2^ (26-41 ml/min/1.73m^2^) and 31 ml/min/1.73m^2^ (23-41 ml/min/1.73m^2^) in linear and non-linear progressors respectively. Both groups also had high degrees of proteinuria, which was reflected in the majority of patients classified with A3 proteinuria.

The categorisation of the nature of the ΔeGFR slope in each patient group was strengthened by a large number of eGFR measurements per patient during the follow-up period with a median of 24 (16–36) in the linear group and 21 (11–37) in the non-linear group. The ΔeGFR itself met the a priori definition of rapid renal decline in both patient groups, and were statistically similar: in linear progressors, the median ΔeGFR was − 5.28 ml/min/1.73m^2^/yr (− 6.75 to − 4.11 ml/min/1.73m^2^/yr) and in non-linear progressors the ΔeGFR was − 4.57 ml/min/1.73m^2^/yr (− 6.46 to − 3.46 ml/min/1.73m^2^/yr); *p*-value of 0.06.

There were however significant differences between the progressor groups with respect to demographic characteristics and co-morbidities. Non-linear patients were typically older and had a higher burden of co-morbidities including diabetes, myocardial infarction, peripheral vascular disease and heart failure. There was also contrasting frequencies of the underlying disease aetiology, most notably seen for autosomal dominant polycystic kidney disease (ADPKD), which was exclusively associated with linear progressors and was the commonest primary renal disease in this group, affecting a quarter of all linear progressor patients.

### Factors associated with ESRD and mortality prior to ESRD

Univariate analyses of the factors associated with ESRD is presented in Additional file 1: Tables S1. In multivariate analysis, younger age, male gender, lack of diabetes, lower eGFR and higher phosphate were significantly associated with progression to ESRD (Table 2).

**Table 2:** Sub-distribution hazard ratios for the competing risks of ESRD and mortality based on a Fine-Gray model

	Mortality prior to ESRD			ESRD		
	Sub-HR	95% CI	p-value	Sub-HR	95% CI	p-value
Age	1.06	1.04–1.08	**< 0.01**	0.97	0.95–0.98	**< 0.01**
Male	1.23	0.72–2.14	0.46	1.49	1.03–2.16	**0.03**
SBP	1.01	0.99–1.02	0.43	1.01	1.00–1.02	0.09
DBP	0.99	0.96–1.01	0.30	1.01	0.99–1.02	0.43
Hypertension	1.27	0.30–5.45	0.75	0.68	0.36–1.30	0.24
Diabetes	1.36	0.78–2.36	0.28	0.63	0.41–0.98	**0.04**
Smoking	1.46	0.82–2.59	0.20	0.89	0.62–1.29	0.55
MI	2.49	1.35–4.61	**0.04**	0.36	0.11–1.15	0.08
CCF	1.08	0.58–2.00	0.81	0.80	0.35–1.83	0.60
Stroke	1.00	0.38–2.64	0.99	0.67	0.30–1.52	0.34
PVD	1.22	0.63–2.33	0.56	0.98	0.53–1.83	0.95
ACEi/ARB	0.63	0.39–1.01	0.06	1.06	0.71–1.58	0.77
Statin	0.79	0.45–1.37	0.40	1.32	0.92–1.91	0.13
eGFR	1.01	0.98–1.03	0.48	0.96	0.94–0.97	**< 0.01**
Bicarbonate	1.00	0.94–1.07	0.89	1.02	0.97–1.08	0.49
Calcium	0.92	0.22–3.87	0.91	0.48	0.15–1.48	0.20
Phosphate	0.38	0.10–1.42	0.15	3.15	1.29–7.67	**0.01**
Albumin	1.00	0.95–1.05	0.83	0.99	0.95–1.02	0.43
Haemoglobin	0.97	0.95–0.99	**0.02**	1.00	0.99–1.01	0.86
A3 proteinuria	0.58	0.34–1.00	0.05	1.00	0.71–1.42	0.99

*Abbreviations*: *SBP* (systolic blood pressure), *DBP* (diastolic blood pressure), *MI* (myocardial infarction), *CCF* (congestive heart failure), *PVD* (peripheral vascular disease), *ACEi* (angiotensin converting enzyme inhibitor), *ARB* (angiotensin receptor blocker), *eGFR-EPI* (eGFR calculated using the EPI-equation)

Three clinical factors were shown to be significantly predictive for mortality prior to ESRD, including older age, history of MI and anaemia (Table 2). Of note, in univariate analyses (Additional file 1: Tables S2), higher SBP, history of diabetes, smoking, PVD, HF and lack of ACEi/ARB use were associated with mortality prior to ESRD but these were found to not be significant once adjusted for other variables.

### Survival analysis comparing linear and non-linear progressors

**Fig. 3:**
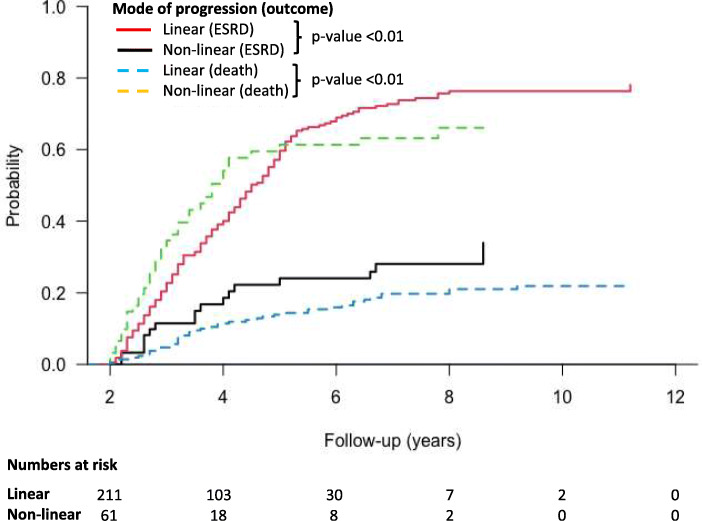
Cumulative incidence functions for ESRD and death prior to ESRD compared between linear and non-linear progressors. Abbreviations: ESRD (end-stage renal disease)

**Fig. 4:**
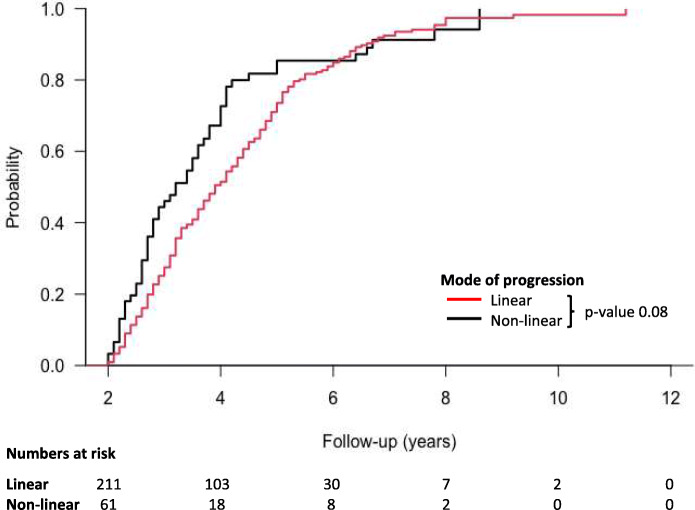
1-Kaplan-Meier curves for probability of survival from the composite outcome of either ESRD or death prior to ESRD compared between linear and non-linear progressors

The study cohort had a median follow-up of 3.7 years (2.8–4.9 years), during which time there were 173 patients who reached ESRD and 81 patients who died prior to ESRD (Table 3). Cumulative incidence function curves (Fig. 3) revealed that rapid linear patients were more likely to develop ESRD compared to their non-linear counterparts (*p*-value< 0.01), but that non-linear progressors suffered higher rates of death prior to ESRD (*p*-value< 0.01). However, when the outcomes were combined as a single endpoint of either ESRD or mortality prior to ESRD (Fig. 4), there was no statistical difference between rapid linear or non-linear progressors (*p*-value 0.08).

**Table 3:** Outcome data

	Rapid linear progressor (n = 211)	Rapid non-linear progressor (n = 61)
ESRD, *n* (%)	156 (76)	17 (28)
Death prior to ESRD, *n* (%)	43 (20)	38 (62)
Under follow-up, *n* (%)	9 (4)	5 (8)
Care transferred to another hospital, *n* (%)	3 (1)	1 (2)

*Abbreviations*: *ESRD* (end-stage renal disease)

## Discussion

This study highlights the differential impact of the pattern of renal trajectory on adverse outcomes in patients with rapidly deteriorating renal function. Patients with rapid linear progression face higher rates of ESRD sooner whilst those with non-linear progression experience higher rates of mortality prior to ESRD.

### Patterns of progression and determinants of adverse outcomes

Our cohort of rapid progressors was predominantly comprised of patients with linear progression, with only 22% of patients deemed to have non-linear progression. This corroborates with work by Weldegiorgis et al. [11], who showed that the majority of the 3523 pooled patients with CKD from six randomised trials demonstrated linear eGFR decline. For instance, they reported a > 50% probability of non-linear progression in 15.1 to 21.2% of patients from non-diabetic kidney trials and 19.3 to 31.7% in diabetic kidney trials.

With respect to the determinants of adverse outcomes, previous work has shown that younger age [12], male gender [13, 14], lower eGFR [7] and higher phosphate [15] are all independently associated with ESRD, and this was borne out in our analysis. In addition, we found that older age [12], a history of MI [16–18], and anaemia [19] are significant determinants of mortality prior to ESRD, and these findings again align with the reported literature. Thus, in our cohort of clearly characterised linear and non-linear progressors, we show the expected overlap of predictive factors important to those who rapidly progress. However, we notably found a lack of diabetes to be predictive for ESRD and also found that A3 proteinuria was not predictive of worse outcomes in our analysis. These findings may likely be attributed to the specific disease characteristics in our population. Indeed, 20% of the entire cohort had ADPKD, of whom 87% reached ESRD. Of the 55 patients with ADPKD only 1 (2%) had diabetes and 8 (15%) were classed as having A3 proteinuria (Additional file 1: Table S3), and this may have affected the impact of diabetes and A3 proteinuria on our outcomes in this cohort.

What is less well known is the association of the pattern of rapid CKD progression, either linear or non-linear, on outcomes such as ESRD and mortality. We hypothesised that rapid linear progressors would fare significantly worse than non-linear progressors, largely as a result of the inevitable culmination of ESRD in linear progressors, whereas potential fluctuating phases of stability may offer a degree of protection to those with non-linear renal decline. We discovered that whilst linear progressors do indeed reach ESRD sooner, non-linear patients were at a significantly greater risk of the competing event of death prior to ESRD.

Phenotypic differences between the two patient groups can help to unravel these findings. For instance, the primary renal disease in 26% of the rapid linear progressors was ADPKD, a condition typically characterised by a linear trajectory to ESRD [20], as a result of the unremitting enlargement of renal cysts that eventually destroy the normal renal architecture.

Furthermore, we found non-linear patients were significantly older, suffered from diabetes and cardiovascular co-morbidities, including MI, PVD and HF. It is conceivable that such patients experience non-linear fluctuations in renal function, including acute kidney injury (AKI), due to uncorrected alterations in their fluid status, as is seen in decompensated heart failure [21]. Additional adjustments to their medications with up-titration of diuretics or with ACEi and ARBs may also cause transient and abrupt eGFR decline. It is clear that the greater burden of cardiovascular disease and the propensity for outpatient AKI-on-CKD events [22] explains the increased mortality experienced by non-linear progressors observed in our cohort.

### Clinical implications

In considering the pattern of CKD progression, we show how two distinct subtypes of rapid progression, linear and non-linear, affect outcomes differentially, which can be explained by differences in patient characteristics. This provides the basis for delivering personalised care to patients, dictated by their disease aetiology, risk factor profile and pattern of CKD progression, and highlights how in some patients the risk of death supersedes the need to prepare for ESRD [6]. This will no doubt influence the communication of risk imparted to patients and help direct the shape of future treatment. Further research in this area is of importance not least because patient heterogeneity for future adverse outcomes is not adequately captured within the current KDIGO staging for CKD, which is based on eGFR and urine albumin:creatinine ratio alone [7]. The future of precision medicine, therefore, relies upon the establishment of improved and refined models that can accurately risk stratify specific CKD patient subgroups.

However, whilst there are specific differences between linear and non-linear progressors with respect to outcomes, there was no survival difference in our analysis between the patients when combining the outcomes as a composite endpoint. Based on this, it is important to emphasise the risk conferred to patients from rapid renal decline per se*,* irrespective of the underlying pattern of progression. In effect, therefore, early identification of rapid progression should prompt close monitoring and aggressive risk factor modification to stabilise and curb a falling eGFR trajectory as best as possible.

Furthermore, this study highlights the significance of estimating ΔeGFR for predicting future outcomes. Indeed, in addition to the last eGFR level, the past eGFR trend has been shown to predict a patient’s future risk of ESRD, especially those with advanced CKD in whom the absolute risk of ESRD is higher [23]. This raises the question as to whether ΔeGFR can be incorporated into current risk prediction tools such as the well-validated Kidney Failure Risk Equation (KFRE), which predicts the 2- and 5-year risk of ESRD in patients with CKD stage 3–5 [15]. However, given that it is equally relevant to reconcile whether death may be more likely than ESRD, the CKD Prognosis Consortium risk calculator provides the 2- and 4-year risk of ESRD as well as the risk of non-fatal cardiovascular events and death prior to ESRD in patients with CKD stage 4, and provides an estimation of the timing of these events in relation to ESRD [24]. Again, whether quantification of the ΔeGFR could improve the risk score in this prediction tool is an area for future research. Further work is also required to determine what time period the ΔeGFR should be assessed over. For instance, does inclusion of a patient’s ΔeGFR calculated over the last year, last 2-years or over a longer time period improve predictive utility over current risk prediction models?

### Strengths and limitations

This study extends our understanding of CKD progression by providing a closer examination of linear and non-linear patterns of rapid progression on future clinical endpoints. It is strengthened by a systematic approach to patient selection that characterises the CKD pattern robustly.

There are also limitations to our work. By its nature of being an observational study, we could not confirm causal association or fully account for unmeasured confounders. In addition, it is a single-centre study with a predominantly Caucasian population and thus the findings may not be generalisable to other patient populations.

## Conclusions

Patients who progress rapidly are exposed to the dual threat of ESRD or mortality prior to ESRD. However, differences in patient characteristics exist between those who progress rapidly in a linear or non-linear fashion, and this has a significant bearing on observed future outcomes. Thus, the phenotypic risk factor profile and individual eGFR trajectory should provide the substrate for personalised therapeutic interventions in this highly vulnerable group of patients in order to deliver optimal CKD care.

## Supplementary Information

**Additional file 1: Table S1.** Univariate analysis using Fine-Gray hazards model to investigate factors associated with ESRD. **Table S2.** Univariate analysis using Fine-Gray hazard model to investigate factors associated with mortality prior to ESRD. **Table S3.** Baseline characteristics of patients with autosomal dominant polycystic kidney disease, diabetic nephropathy and glomerulonephritis.

## Data Availability

The data analysed during the current study are available from the corresponding author on reasonable request.

## Abbreviations

ACEi: Angiotensin converting enzyme inhibitor
AKI: Acute kidney injury
ARB: Angiotensin receptor blocker
ADPKD: Autosomal dominant polycystic kidney disease
CKD: Chronic kidney disease
CI: Confidence interval
ΔeGFR: Delta estimated glomerular filtration rate
DM: Diabetes mellitus
DBP: Diastolic blood pressure
ESRD: End-stage renal disease
eGFR: Estimated glomerular filtration rate
Hb: Haemoglobin
HF: Heart failure
MI: Myocardial infarction
PVD: Peripheral vascular disease
SBP: Systolic blood pressure
SKS: Salford Kidney Study
uPCR: Urine protein:creatinine ratio
