# Supplementary material for: Adverse outcomes associated with rapid linear and non-linear patterns of chronic kidney disease progression
Source: BMC Nephrol. 2021 Mar 6;22:82. doi: 10.1186/s12882-021-02282-5 (PMC7937251; doi:10.1186/s12882-021-02282-5)
Supplement: Supplementary file 1 — Additional file 1: Table S1. Univariate analysis using Fine-Gray hazards model to investigate factors associated with ESRD. Table S2. Univariate analysis using Fine-Gray hazard model to investigate factors associated with mortality prior to ESRD. Table S3. Baseline characteristics of patients with autosomal dominant polycystic kidney disease, diabetic nephropathy and glomerulonephritis. [file 12882_2021_2282_MOESM1_ESM.docx]

**Adverse outcomes associated with rapid linear and non-linear patterns of chronic kidney disease progression**

Ibrahim Ali, Rajkumar Chinnadurai, Sara T. Ibrahim, Philip A. Kalra

**Table S1** Univariate analysis using Fine-Gray hazards model to investigate factors associated with ESRD.

| **Variable** | **Univariate model**  **HR (95% CI)** | **p-value** |
| --- | --- | --- |
| Age (per year) | 0.97 (0.96-0.98) | **<0.01** |
| Male | 0.93 (0.69-1.24) | 0.61 |
| Systolic blood pressure (per 1mmHg) | 0.99 (0.99-1.00) | 0.10 |
| Diastolic blood pressure (per 1mmHg) | 1.02 (1.01-1.04) | **<0.01** |
| Hypertension | 0.60 (0.35-1.01) | 0.05 |
| Diabetes mellitus | 0.60 (0.43-0.85) | **<0.01** |
| Past/current smoking history | 0.80 (0.60-1.07) | 0.14 |
| Myocardial infarction | 0.22 (0.08-0.61) | **<0.01** |
| Peripheral vascular disease | 0.59 (0.34-1.03) | 0.06 |
| Stroke | 0.72 (0.37-1.39) | 0.33 |
| Heart failure | 0.40 (0.19-0.82) | **0.01** |
| ACEi/ARB | 1.17 (0.82-1.67) | 0.39 |
| Statin | 0.81 (0.61-1.09) | 0.17 |
| eGFR (per 1ml/min/1.73m^2^) | 0.98 (0.96-0.99) | **<0.01** |
| Bicarbonate (per 1mmol/L) | 0.96 (0.92-1.01) | 0.13 |
| Calcium (per 0.1mmol/L) | 0.55 (0.20-1.51) | 0.25 |
| Phosphate (per 0.1mmol/L) | 3.57 (1.64-7.78) | **<0.01** |
| Albumin (per 1g/L) | 1.00 (0.96-1.04) | 0.98 |
| Haemoglobin (per 1g/L) | 0.99 (0.98-1.01) | 0.78 |
| A3 proteinuria | 1.36 (1.01-1.83) | **0.04** |

**Abbreviations**: ACEi/ARB (angiotensin converting enzyme inhibitor/ angiotensin receptor blocker use); eGFR (estimated glomerular filtration rate)

**Table S2** Univariate analysis using Fine-Gray hazard model to investigate factors associated with mortality prior to ESRD.

| **Variable** | **Univariate model**  **HR (95% CI)** | **p-value** |
| --- | --- | --- |
| Age (per year) | 1.07 (1.05-1.09) | **<0.01** |
| Male | 1.32 (0.86-2.03) | 0.21 |
| Systolic blood pressure (per 1mmHg) | 1.02 (1.01-1.03) | **<0.01** |
| Diastolic blood pressure (per 1mmHg) | 0.98 (0.97-0.99) | **0.03** |
| Hypertension | 3.04 (0.33-0.76) | 0.12 |
| Diabetes mellitus | 2.32 (1.51-3.57) | **<0.01** |
| Past/current smoking history | 1.59 (0.99-2.55) | 0.05 |
| Myocardial infarction | 3.70 (1.96-6.99) | **<0.01** |
| Peripheral vascular disease | 2.69 (1.57-4.61) | **<0.01** |
| Stroke | 1.93 (0.93-3.98) | 0.08 |
| Heart failure | 3.56 (2.13-5.96) | **<0.01** |
| ACEi/ARB | 0.61 (0.39-0.97) | **0.04** |
| Statin | 1.53 (0.97-2.42) | 0.07 |
| eGFR-EPI (per 1ml/min/1.73m^2^) | 0.99 (0.98-1.02) | 0.65 |
| Bicarbonate (per 1mmol/L) | 1.02 (0.96-1.08) | 0.58 |
| Calcium (per 0.1mmol/L) | 1.56 (0.36-6.67) | 0.55 |
| Phosphate (per 0.1mmol/L) | 0.43 (0.16-1.14) | 0.09 |
| Albumin (per 1g/L) | 0.98 (0.95-1.01) | 0.25 |
| Haemoglobin (per 1g/L) | 0.98 (0.97-0.99) | 0.02 |
| A3 proteinuria | 0.75 (0.48-1.15) | 0.18 |

**Abbreviations**: ACEi/ARB (angiotensin converting enzyme inhibitor/ angiotensin receptor blocker use); eGFR (estimated glomerular filtration rate)

**Table S3** Baseline characteristics of patients with autosomal dominant polycystic kidney disease, diabetic nephropathy and glomerulonephritis.

| **Variable** | **ADPKD**  **(n=55)** | **Diabetic nephropathy**  **(n=67)** | **Glomerulonephritis**  **(n=34)** |
| --- | --- | --- | --- |
| Age (years | 50.7 (45.5-56.4) | 63.2 (54.7-73.2) | 54.8 (39.4-64.0) |
| Men, *n* (%) | 19 (35) | 45 (67) | 19 (56) |
| Caucasian, *n* (%) | 45 (82) | 60 (90) | 27 (79) |
| Systolic blood pressure (mmHg) | 135 (124-146) | 145 (138-160) | 139 (132-157) |
| Diastolic blood pressure (mmHg) | 80 (76-88) | 75 (70-80) | 78 (70-84) |
| Hypertension, *n* (%) | 50 (91) | 66 (99) | 34 (100) |
| Diabetes, *n* (%) | 1 (2) | 67 (100) | 5 (15) |
| Past/current smoking history, *n* (%) | 31 (56) | 45 (67) | 23 (68) |
| Myocardial infarction, *n* (%) | 0 (0) | 14 (21) | 0 (0) |
| Peripheral vascular disease, *n* (%) | 2 (36) | 14 (21) | 2 (6) |
| Stroke, *n* (%) | 3 (5) | 8 (12) | 1 (3) |
| Heart failure, *n* (%) | 1 (2) | 14 (21) | 2 6) |
| ACEi/ARB, *n* (%) | 40 (73) | 56 (84) | 32 (94) |
| Statin, *n* (%) | 21 (38) | 55 (82) | 22 (65) |
| Years follow-up | 4.3 (3.2-5.1) | 3.4 (2.7-4.3) | 3.6 (2.7-5.3) |
| **Laboratory results** |  |  |  |
| eGFR-EPI (ml/min/1.73m^2^) | 35 (25-44) | 33 (27-41) | 39 (34-46) |
| eGFR measurements, *n* | 21 (16-31) | 26 (13-40) | 27 (18-42) |
| ΔGFR (±ml/min/1.73m^2^/yr) | -5.64 (-6.75 to -4.18) | -4.76 (-6.41 to -3.59) | -6.36 (-8.80 to -5.00) |
| Bicarbonate (mmol/L) | 23.0 (20.9-25.0) | 23.0 (21.2-25.3) | 22.0 (20.3-25.2) |
| Urea (mmol/L) | 11.8 (9.8-16.5) | 12.5 (10.3-17.3) | 11.9 (9.5-16.4) |
| Calcium (mmol/L) | 2.31 (2.24-2.36) | 2.31 (2.25-2.41) | 2.29 (2.21-3.37) |
| Phosphate (mmol/L) | 1.15 (1.04-1.30) | 1.17 (1.05-1.34) | 1.16 (1.03-1.28) |
| Albumin (g/L) | 45 (42-46) | 40 (37-43) | 40 (35-42) |
| Total cholesterol/HDL ratio | 3.41 (2.67-4.04) | 3.27 (2.65-4.17) | 3.96 (3.19-4.46) |
| Haemoglobin (g/L) | 125 (117-135) | 118 (108-125) | 129 (116-138) |
| Urine protein:creatinine ratio (g/mol) | 22 (15-41) | 171 (54-351) | 179 (100-490) |
| A1 proteinuria (<15g/mol), *n* (%) | 13 (24) | 3 (4) | 0 (0) |
| A2 proteinuria (15-50g/mol), *n* (%) | 34 (62) | 13 (19) | 5 (15) |
| A3 proteinuria (>50g/mol), *n* (%) | 8 (15) | 51 (76) | 29 (85) |
| **Outcomes** |  |  |  |
| ESRD, *n* (%) | 48 (87) | 35 (52) | 23 (68) |
| Mortality prior to ESRD, *n* (%) | 2 (4) | 30 (45) | 8 (24) |
| Under follow-up, *n* (%) | 5 (9) | 2 (3) | 3 (9) |

**Abbreviations**: ACEi/ARB (angiotensin converting enzyme inhibitor/ angiotensin receptor blocker use); eGFR (estimated glomerular filtration rate); ESRD (end-stage renal disease)
